# The Size-Value Compatibility Effect

**DOI:** 10.1038/s41598-020-62419-8

**Published:** 2020-03-25

**Authors:** Kunihiro Hasegawa

**Affiliations:** National Institute of Advanced Industrial Science and Technology (AIST), Department of Information Technology and Human Factors, Tsukuba, 305-8566 Japan

**Keywords:** Psychology, Human behaviour

## Abstract

This study aimed to examine the relationship between an object’s physical size and judgements of its value. Two preregistered experiments were performed to explore a size-value compatibility effect. Two images of Japanese-yen coins with different values but similar actual sizes (10-yen and 100-yen) were manipulated for size and presented side-by-side on a computer screen. Participants judged which coin was larger or smaller based on the images. Results revealed that size judgements were slower and less accurate when the lower-value coin was presented as larger than the higher-value coin, compared to when the lower-value coin was presented as smaller. This effect was observed even after participants had been allowed to examine the physical coins prior to the experiment to judge their actual size. This finding suggests that participants perceived the coins’ values based on their sizes, indicating it may be difficult for many people to stop thinking ‘better is bigger’.

## Introduction

For over a half of century, research has indicated that individual perception and cognition of an object may be affected by its value. In a classic experiment, ten-year-old children were asked to judge the size of coins, and their assessments were shown to be dependent on the participant’s economic status^1^. Specifically, compared to rich children, poor children tended to overestimate the size of higher-value coins. Further, Kahneman and Tversky reported on the heuristics people often use to make unintentional judgements based on simple information, such as physical size, especially when they are uncertain^2,3^. Later, the process of the unintentional (intuitive) judgement was called System-1, and was differentiated from the process of controlled (reflective) judgement, or System-2^4^. Following this concept, empirical evidence has demonstrated the relationship between physical size and value in terms of consumers’ perceptions of price information^5^, positive connotation of words^6^, and aesthetic preferences^7^. These findings suggest that people may tend to think that ‘bigger is better/better is bigger’.

**Figure 1:**
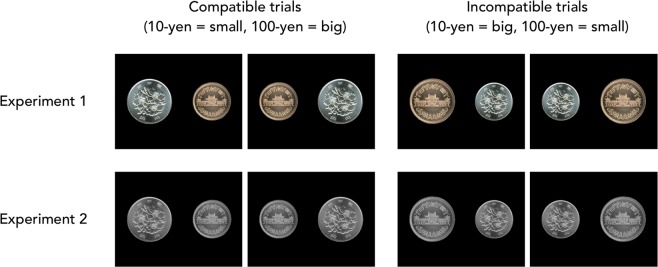
All patterns of the stimuli in the present study. For compatible trials (left panels), the higher-value coin (100-yen) was presented as larger, and the lower-value coin (10-yen) was presented as smaller. For incompatible trials (right panels), the higher-value coin was presented as smaller, and the lower-value coin was presented as larger. The photos were taken from Wikipedia [https://www.wikipedia.org], and are free-use media [https://commons.wikimedia.org].

However, these previous studies depended on subjective reports regarding the size-value relationship. Thus, the possibility remained that participants’ responses in the previous studies were made reflectively. Therefore, there was still uncertainty whether the tendency of thinking ‘bigger is better/better is bigger’ is intentional or unintentional. The present study identified a new phenomenon around unintentional size-based value judgements using a modified version of the visual size-based Stroop task^8^. In the experiments, two images were presented, and participants were asked to judge which image was larger or smaller on the screen. In the original size-based Stroop task, in trials evaluating images ‘compatible’ with the size-value notion, an image of a large object (e.g. elephant) was presented as larger than an image of a small object (e.g. mouse); for ‘incompatible’ trials, the sizes of the images were reversed. As a result, participants’ reaction times were longer and their responses were less accurate for incompatible compared to compatible trials. This showed the size-size compatibility effect between objects’ actual sizes and their ‘visual’ sizes, as presented under experimental conditions. In the present study, two images of Japanese-yen coins with different values but similar actual sizes (10-yen and 100-yen) were manipulated for size and presented side-by-side on a computer screen (Fig. 1). For compatible trials, an image of a 100-yen coin was presented as larger than an image of a 10-yen coin, and for incompatible trials, the sizes of the coin images were reversed. Importantly, these coins have different values, but are similar in actual size, as the diameter of the 10-yen coin (23.5 mm) is only slightly larger than the 100-yen coin (22.6 mm). Therefore, if the compatibility effect were observed in the present study, it would indicate the size-value compatibility effect, not the size-size compatibility effect.

Additionally, Experiment 2 was conducted to eliminate the effects of the size illusion based on objects’ colour. In Experiment 1, there were differences in colour and/or contrast between the coin images. Therefore, the colour/contrast differences may have affected size judgements in Experiment 1, as a previous study showed the possibility that colour and contrast differences could reduce the size illusion^9^. For this aim, greyscale images were used as stimuli in Experiment 2 (Fig. 1). Further, a larger number of participants (twice the sample size of Experiment 1) were invited to participate in Experiment 2, as large sample size studies can come closer to a true effect size.

## Results

### Experiment 1

The results are summarized in Table 1. Error trials and trials in which reaction time (RT) was shorter than 200 ms or longer than 1,500 ms were excluded from calculations of mean RTs for each participant (1.6% of all data in the larger task and 1.3% of all data in the smaller task). The threshold for outliers was set to match a previous study using the size Stroop task^8^, and had been preregistered before the start of the experiment (see the open practices statement in Methods section).

**Table 1:** Summary of results in Experiment 1.

	Percentages of errors (PEs)				Reaction times (RTs; ms)			
	Compatible		Incompatible		Compatible		Incompatible	
	Mean	(SD)	Mean	(SD)	Mean	(SD)	Mean	(SD)
*Larger task*								
Overall	0.9	(1.4)	4.0	(3.8)	506.9	(89.4)	571.2	(102.8)
Block 1	1.3	(2.7)	4.9	(6.7)	523.5	(117.0)	582.5	(132.3)
Block 2	0.6	(1.7)	4.7	(7.8)	502.8	(75.1)	565.7	(79.2)
Block 3	0.6	(1.7)	2.9	(4.2)	501.7	(99.5)	575.4	(121.4)
Block 4	1.3	(3.1)	3.4	(4.2)	509.3	(92.8)	571.5	(122.8)
Block 5	0.6	(1.7)	4.2	(5.3)	498.8	(91.5)	561.8	(96.4)
*Smaller task*								
Overall	2.0	(1.9)	4.6	(3.4)	530.5	(81.8)	586.6	(97.1)
Block 1	2.9	(3.3)	6.1	(6.6)	533.4	(90.7)	589.5	(119.2)
Block 2	2.1	(3.6)	5.0	(4.4)	524.1	(81.5)	576.4	(112.2)
Block 3	2.5	(3.9)	5.5	(4.7)	520.7	(84.6)	580.5	(116.1)
Block 4	1.1	(2.6)	3.0	(4.2)	521.6	(83.0)	578.5	(99.4)
Block 5	1.5	(2.4)	3.6	(4.7)	553.5	(116.0)	608.1	(116.3)

#### Larger task

The data were analysed by a paired samples *t*-test using the factor of compatibility (compatible or incompatible). The mean percentages of errors (PEs) for compatible trials were significantly lower than PEs for incompatible trials, *t*(23) = 4.40, *p* < .001, 95% CI = [1.63, 4.51], *d* = 1.08. The mean RTs for compatible trials were significantly shorter than RTs for incompatible trials, *t*(23) = 9.13, *p* < .001, 95% CI = [49.74, 78.87], *d* = 0.67.

Furthermore, PEs and RTs in each block of the larger task were additionally analysed by a two-way repeated measure analysis of variance (ANOVA) for the factors of compatibility (compatible or incompatible) and block (1, 2, 3, 4, or 5). The ANOVA for PEs suggested that the main effect of compatibility was significant, *F*(1, 23) = 17.89, *p* < .001, η_G_^2^ = 0.12. The main effect of block was not significant, *F*(2.2, 49.9) = 0.91, *p* = .464, η_G_^2^ = 0.01. In this analysis, degrees of freedom were corrected using the Greenhouse-Geisser method, because Mauchly’s test of sphericity indicated that the assumption of sphericity was violated, Mauchly’s *W* = 0.24, *p* < .001, Greenhouse-Geisser ε = 0.54. These factors’ interaction was not significant, *F*(2.6, 60.1) = 0.65, *p* = .630, η_G_^2^ = 0.01. In this analysis, degrees of freedom were corrected using the Greenhouse-Geisser method, because Mauchly’s test of sphericity indicated that the assumption of sphericity was violated, Mauchly’s *W* = 0.30, *p* = .002, Greenhouse-Geisser ε = 0.65. The ANOVA for RTs suggested that the main effect of compatibility was significant, *F*(1, 23) = 84.64, *p* < .001, η_G_^2^ = 0.09. The main effect of block was not significant, *F*(2.8, 65.3) = 1.29, *p* = .281, η_G_^2^ < 0.01. In this analysis, degrees of freedom were corrected using the Greenhouse-Geisser method, because Mauchly’s test of sphericity indicated that the assumption of sphericity was violated, Mauchly’s *W* = 0.39, *p* = .017, Greenhouse-Geisser ε = 0.71. The interaction of these factors was not significant, *F*(4, 92) = 0.36, *p* = .839, η_G_^2^ < 0.01.

#### Smaller task

The data were analysed by a paired samples *t*-test using the factor of compatibility (compatible or incompatible). PEs for compatible trials were significantly lower than PEs for incompatible trials, *t*(23) = 3.81, *p* < .001, 95% CI = [1.20, 4.04], *d* = 0.95. RTs for compatible trials were significantly shorter than RTs for incompatible trials, *t*(23) = 4.85, *p* < .001, 95% CI = [32.15, 80.05], *d* = 0.62.

Furthermore, PEs and RTs in each block of the smaller task were additionally analysed using an ANOVA for the factors of compatibility (compatible or incompatible) and block (1, 2, 3, 4, or 5). The ANOVA for PEs suggested that the main effect of compatibility was significant, *F*(1, 23) = 14.77, *p* < .001, η_G_^2^ = 0.09. The main effect of block was significant, *F*(4, 92) = 4.62, *p* = .002, η_G_^2^ = 0.05. These factors’ interaction was not significant, *F*(4, 92) = 0.27, *p* = .894, η_G_^2^ < 0.01. The ANOVA for RTs suggested that the main effect of compatibility was significant, *F*(1, 23) = 22.69, *p* < .001, η_G_^2^ = 0.07. The main effect of block was not significant, *F*(4, 92) = 2.12, *p* = .085, η_G_^2^ = 0.01. These factors’ interaction was not significant, *F*(4, 92) = 0.04, *p* = .996, η_G_^2^ < 0.01.

### Experiment 2

The results are summarized in Table 2. Error trials and trials in which RT was shorter than 200 ms or longer than 1,500 ms were excluded from calculations of mean RTs for each participant (1.0% of all data in the larger task and 1.6% of all data in the smaller task). The threshold of outliers was set to match Experiment 1, and had been preregistered before the start of the experiment (see the open practices statement in Methods section).

**Table 2:** Summary of results in Experiment 2.

	Percentages of errors (PEs)				Reaction times (RTs; ms)			
	Compatible		Incompatible		Compatible		Incompatible	
	Mean	(SD)	Mean	(SD)	Mean	(SD)	Mean	(SD)
*Larger task*								
Overall	1.0	(1.4)	2.6	(2.2)	456.0	(70.5)	516.1	(89.7)
Block 1	0.4	(1.4)	2.7	(3.0)	471.2	(86.3)	536.9	(107.9)
Block 2	1.2	(2.1)	2.2	(3.9)	454.6	(77.2)	503.6	(90.4)
Block 3	1.5	(2.9)	2.2	(3.3)	448.3	(73.2)	509.8	(94.0)
Block 4	1.4	(3.1)	3.3	(4.4)	453.1	(84.2)	520.1	(109.3)
Block 5	0.7	(2.2)	2.4	(3.3)	453.3	(68.1)	514.0	(115.0)
*Smaller task*								
Overall	1.4	(2.0)	4.1	(3.3)	484.7	(76.6)	546.0	(112.2)
Block 1	1.7	(3.0)	4.4	(5.1)	485.6	(95.8)	549.7	(131.0)
Block 2	1.1	(2.6)	4.7	(5.3)	492.6	(96.7)	541.4	(137.1)
Block 3	1.4	(2.8)	3.8	(5.2)	469.6	(75.0)	535.7	(111.1)
Block 4	1.3	(2.9)	3.8	(4.8)	483.1	(85.1)	547.8	(128.5)
Block 5	1.4	(3.1)	3.6	(4.3)	492.9	(87.5)	556.5	(119.4)

#### Larger task

The data were analysed by a paired samples *t*-test using the factor of compatibility (compatible or incompatible). PEs for compatible trials were significantly lower than PEs for incompatible trials, *t*(47) = 4.70, *p* < .001, 95% CI = [0.88, 2.20], *d* = 0.81. RTs for compatible trials were significantly shorter than RTs for incompatible trials, *t*(47) = 10.97, *p* < .001, 95% CI = [49.06, 71.08], *d* = 0.74.

Furthermore, PEs and RTs in each block of the larger task were additionally analysed using an ANOVA for the factors of compatibility (compatible or incompatible) and block (1, 2, 3, 4, or 5). The ANOVA for PEs suggested that the main effect of compatibility was significant, *F*(1, 47) = 21.39, *p* < .001, η_G_^2^ = 0.06. The main effect of block was not significant, *F*(3.4, 159.5) = 1.62, *p* = .170, η_G_^2^ < 0.01. In this analysis, degrees of freedom were corrected using the Greenhouse-Geisser method, because Mauchly’s test of sphericity indicated that the assumption of sphericity was violated, Mauchly’s *W* = 0.69, *p* = .047, Greenhouse-Geisser ε = 0.85. These factors’ interaction was not significant, *F*(4, 188) = 1.42, *p* = .228, η_G_^2^ < 0.01. The ANOVA for RTs suggested that the main effect of compatibility was significant, *F*(1, 47) = 109.71, *p* < .001, η_G_^2^ = 0.10. The main effect of block was significant, *F*(2.7, 126.4) = 3.48, *p* = .009, η_G_^2^ < 0.01. In this analysis, degrees of freedom were corrected using the Greenhouse-Geisser method, because Mauchly’s test of sphericity indicated that the assumption of sphericity was violated, Mauchly’s *W* = 0.44, *p* < .001, Greenhouse-Geisser ε = 0.67. These factors’ interaction was not significant, *F*(3.1, 146.5) = 1.02, *p* = .399, η_G_^2^ < 0.01. In this analysis, degrees of freedom were corrected using the Greenhouse-Geisser method, because Mauchly’s test of sphericity indicated that the assumption of sphericity was violated, Mauchly’s *W* = 0.61, *p* = .008, Greenhouse-Geisser ε = 0.78.

#### Smaller task

The data were analysed by a paired samples *t*-test using the factor of compatibility (compatible or incompatible). PEs for compatible trials were significantly lower than PEs for incompatible trials, *t*(47) = 6.33, *p* < .001, 95% CI = [1.84, 3.56], *d* = 1.00. RTs for compatible trials were significantly shorter than RTs for incompatible trials, *t*(47) = 9.28, *p* < .001, 95% CI = [48.05, 74.64], *d* = 0.64.

Furthermore, PEs and RTs in each block of the smaller task were additionally analysed using an ANOVA for the factors of compatibility (compatible or incompatible) and block (1, 2, 3, 4, or 5). The ANOVA for PEs suggested that the main effect of compatibility was significant, *F*(1, 47) = 39.16, *p* < .001, η_G_^2^ = 0.10. The main effect of block was not significant, *F*(4, 188) = 0.56, *p* = .690, η_G_^2^ < 0.01. These factors’ interaction was not significant, *F*(4, 188) = 0.65, *p* = .629, η_G_^2^ < 0.01. The ANOVA for RTs suggested that the main effect of compatibility was significant, *F*(1, 47) = 86.76, *p* < .001, η_G_^2^ = 0.08. The main effect of block was not significant, *F*(3.0, 141.3) = 1.21, *p* = .310, η_G_^2^ < 0.01. In this analysis, degrees of freedom were corrected using the Greenhouse-Geisser method, because Mauchly’s test of sphericity indicated that the assumption of sphericity was violated, Mauchly’s *W* = 0.53, *p* = .001, Greenhouse-Geisser ε = 0.75. These factors’ interaction was not significant, *F*(4, 188) = 0.98, *p* = .417, η_G_^2^ < 0.01.

## Discussion

The findings of the present study indicated that people consider an object’s value when judging its physical size. To demonstrate this, two experiments using a coin-size modified Stroop task were conducted. The results of Experiment 1 showed that participants took more time and were less accurate in incompatible than compatible trials. Furthermore, the compatibility effect was robustly reproduced in Experiment 2, using a double sample size, even though the effect of colour/contrast on the size judgement of an image had been excluded. This implies that the effect was caused by size-value compatibility.

In studies using the original version of the physical size Stroop effect^8^, it was argued that when people recognize an object, they unintentionally judge how big it would be in the real world. Similarly, when people recognize a higher-value object, they may unintentionally assume it is also bigger. Thus, the size-value compatibility effect in the present study may reflect that people strongly associate physical size with value. This is consistent with the concept of Kahneman’s System-1^4^.

Furthermore, this unintentionality was also suggested by block-wise analyses. There were only four patterns of coin image combinations in the present study (see Fig. 1), and each pattern was repeatedly presented 100 times in total, per participant. If the size-value compatibility effect was caused by reflective judgement, the effect should vanish when participants consciously understood that size-based value judgements were not necessary in the present task, as the trials were repeated. However, the size-value compatibility effect was consistent throughout the experiment. This suggests that size-based value judgements may have been made unintentionally, even if participants fully understood they were not necessary in the present task. Thus, it may be difficult for many people to stop thinking ‘better is bigger’.

## Methods

### Ethics statement

This research complied with the Declaration of Helsinki and was approved by the Institutional Review Board at the National Institute of Advanced Industrial Science and Technology (AIST). Informed consent was obtained from each participant.

### Open practices statement

The preregistrations for Experiments 1 and 2 can be accessed at *AsPredicted* [http://aspredicted.org/blind.php?x = 4k7b3j] and [http://aspredicted.org/blind.php?x = 935az6]. De-identified data and data analysis scripts are posted at *Open Science Framework* [https://osf.io/5qwvg/]. Access to the data is limited to qualified researchers. The materials used in this study are widely available.

### Participants

Twenty-four Japanese (15 females and 9 males, mean age = 22.92 years old) participated in Experiment 1, and another 48 Japanese (23 females and 25 males, mean age = 22.50 years old) participated in Experiment 2. All reported normal or corrected-to-normal vision. Sample sizes in Experiments 1 and 2 were decided based on the effect size in a preliminary experiment, using the same procedure as the smaller task in the present experiment (*N* = 13). The estimated sample size using G*Power3 (http://www.gpower.hhu.de) was *N* = 12 (paired *t*-test, tails = 2, effect size = 1.17, power = 0.95). However, studies on the Stroop effect have usually recruited more than 20 participants. Therefore, 24 young adults were recruited for Experiment 1. Additionally, 48 young adults were recruited for Experiment 2, as a large sample size can yield more accurate effect sizes.

### Apparatus and stimuli

The experiment was conducted using Python 3.6.6 (https://www.python.org) with PsychoPy 3.1.3^10–12^, which was controlled by a computer with a central processing unit (Intel, Core i7-6700) and a graphics processing unit (Nvidia, Quadro P400). The visual stimuli were presented on a high-speed liquid crystal display monitor (BenQ, XL2540), which was set to a 1,980 × 1,080 resolution, with a 60-Hz refresh rate. The viewing distance was 57 cm. Participants’ heads were kept in a fixed position using a chin rest. Responses were entered using a keyboard (Corsair, K63).

Images of 10- and 100-yen coins were used as the stimuli in all experiments. The images were taken from Wikipedia [https://www.wikipedia.org], and were free-use media [https://commons.wikimedia.org]. Images of the ‘tails’ side of the coins were used, and indications of the coins’ values were erased using image processing. All stimuli patterns are shown in Fig. 1. In Experiment 2, greyscale versions of the stimuli were used. The brightness of greyscale images was normalized (mean brightness = 96 and standard deviation of brightness = 32 in 8-bit colour system) using OpenCV (https://opencv.org) in Python.

The stimuli images were presented side-by-side, and the centre of each image was located at ±5 degrees of visual angle away from the centre of the screen. The sizes of the images on the screen were manipulated. The larger one was presented at 4.0 degrees of visual angle, and the smaller one was presented at 3.6 degrees of visual angle.

### Design and procedures

Each experiment consisted of 10 experimental blocks, each block included 20 compatible and 20 incompatible trials, and the trial sequence of each block was randomized using the Mersenne Twister method. For compatible trials, a 10-yen coin was presented as smaller and a 100-yen coin was presented larger, relative to each other. For incompatible trials, a 10-yen coin was presented as larger and a 100-yen coin was presented as smaller. In one half of the experiment, participants were asked to judge which image was visually larger (‘larger task’); in the other half, participants were asked to judge which image was visually smaller (‘smaller task’). Half of the participants executed the larger task before the smaller task, while this order was reversed for the other half of the participants.

Before starting the experiment, participants physically held the coins to verify their actual sizes. The reason for this was that the mental size effect must be addressed in advance, as a factor of concern. If participants remembered the 100-yen coin as larger than the 10-yen coin, this inaccuracy would interfere with the size judgements of coin images on the screen. Thus, the present study was designed so that participants could touch actual coins for a size comparison before starting the experiment, and all participants were aware there is almost no size difference between these two coins.

In all trials, a fixation cross was initially presented for 700 ms. Then, the visual stimuli were presented until participants’ responses were registered. Participants were asked to judge as quickly and accurately as possible which image was larger/smaller on the screen by using their left index finger to press the ‘F’ key if the image on the left was larger/smaller, and using their right index finger to press the ‘J’ key if the image on the right was larger/smaller. A blank screen was presented for 900 ms between each trial. Participants could rest during the intervals between experimental blocks.

## References

[1] Bruner JS, Goodman CC (1947). Value and need as organizing factors in perception. J. Abnorm. Psychol..

[2] Kahneman D, Tversky A (1973). On the psychology of prediction. Psychol. Rev..

[3] Tversky A, Kahneman D (1974). Judgment under Uncertainty: Heuristics and Biases. Science.

[4] Kahneman, D. & Frederick, S. In *Heuristics and Biases* (eds. Gilovich, T., Griffin, D. & Kahneman, D.) 49–81 (Cambridge University Press, 2002).

[5] Coulter KS, Coulter RA (2005). Size does matter: The effects of magnitude representation congruency on price perceptions and purchase likelihood. J. Consum. Psychol..

[6] Meier BP, Robinson MD, Caven AJ (2008). Why a big mac is a good mac: Associations between affect and size. Basic. Appl. Soc. Psychol..

[7] Silvera DH, Josephs RA, Giesler RB (2002). Bigger is better: the influence of physical size on aesthetic preference judgments. J. Behav. Decis. Mak..

[8] Konkle T, Oliva A (2012). A familiar-size Stroop effect: real-world size is an automatic property of object representation. J. Exp. Psychol. Hum. Percept. Perform..

[9] Zhang X, Qian J, Liang Q, Huang Z (2018). Effects of Color and Luminance Contrast on Size Perception-Evidence from a Horizontal Parallel Lines Illusion. Vision.

[10] Peirce JW (2007). PsychoPy—Psychophysics software in Python. J. Neurosci. Meth.

[11] Peirce J (2019). PsychoPy2: Experiments in behavior made easy. Behav. Res. Methods.

[12] Peirce JW (2008). Generating stimuli for neuroscience using PsychoPy. Front. Neuroinform.

